# Supplementary material for: Clusternomics: Integrative context-dependent clustering for heterogeneous datasets
Source: PLoS Comput Biol. 2017 Oct 16;13(10):e1005781. doi: 10.1371/journal.pcbi.1005781 (PMC5658176; doi:10.1371/journal.pcbi.1005781)
Supplement: S1 Appendix — Details on the model, implementation, algorithm setting and MCMC convergence. (PDF) [file pcbi.1005781.s001.pdf]

Clusternomics: Integrative Context-Dependent Clustering for Heterogeneous Datasets
S1 Appendix: Supplementary materials

Contents

S1.1 Model details **3**
S1.1.1 Generative process in the decoupled model 3
S1.1.2 Relation to the hierarchical Dirichlet process and other models 3

S1.2 Inference in the context-dependent clustering model **6**
S1.2.1 Posterior inference in the fully combinatorial Clusternomics model 6
S1.2.2 Posterior inference in the decoupled Clusternomics model 9
S1.2.3 Variational inference in the fully combinatorial model 11

S1.3 Implementation and results **13**
S1.3.1 Clusternomics and ad-hoc integrative clustering 13
S1.3.2 MCMC sampling and runtime 14
S1.3.3 Parameter settings 15
S1.3.3.1 Simulated datasets 15
S1.3.3.2 Cancer subtyping 16
S1.3.4 Stability of results: posterior co-clustering matrices 16
S1.3.5 Choosing number of clusters 16

S1.1 Model details

Here we provide additional details regarding the context-dependent clustering model described in the main paper.

S1.1.1 Generative process in the decoupled model

To illustrate workings of the second formulation of the clustering model, the decoupled model, here we give an overview of the generative process in the model:

1. For contexts $c = 1, \ldots, C$, sample parameters for $K^{(c)}$ clusters from the prior

$$\theta_l^{(c)} \sim \boldsymbol{H}^{(c)}$$

2. For contexts $c = 1, \ldots, C$, sample local mixture proportions

$$\boldsymbol{\pi}^{(c)} \sim \text{Dirichlet}\left(\frac{\alpha_0}{K^{(c)}}, \ldots, \frac{\alpha_0}{K^{(c)}}\right)$$

3. Sample global mixture proportions for S global clusters

$$\boldsymbol{\rho} \sim \text{Dirichlet}\left(\frac{\gamma_0}{S}, \ldots, \frac{\gamma_0}{S}\right)$$

4. For the S global clusters, sample assignment to local clusters in each context

$$k_s^{(c)} \mid \pi^{(c)} \;\sim\; \text{Categorical}\left(\pi^{(c)}\right), c = 1, \ldots, C$$

5. For N data points, sample their assignment to global clusters

$$z_n \mid \rho \;\sim\; \text{Categorical}\left(\rho\right)$$

6. Sample value of each data point given its cluster assignment

$$\boldsymbol{x}_n^{(c)} \mid z_n, \left(k_s^{(c)}\right)_{s=1}^{S}, (\theta_l)_{l=1}^{K^{(c)}} \;\sim\; F^{(c)}\left(\boldsymbol{x}_n^{(c)} \mid \theta_{k_{z_n}}^{(c)}\right)$$

S1.1.2 Relation to the hierarchical Dirichlet process and other models

Both models presented in the main paper are finite models with pre-specified numbers of clusters $K^{(c)}$ for each context c and the number of global clusters S. However, we can also view them as finite approximations to the Dirichlet process mixture model.

The decoupled model (Eq. (5) in the main paper) is composed of two levels of finite Dirichlet mixture models. Ishwaran and Zarepour [1, 2] show that a mixture model with a finite Dirichlet prior and with a symmetric concentration parameter is a close approximation for the Dirichlet process. More precisely, the finite Dirichlet prior converges weakly (converges in distribution) to a Dirichlet process as the number of clusters goes to infinity, $K \to \infty$ [2]. This applies to both Dirichlet distributions in the decoupled model (Eq. (1)).

Kurihara et al. [3] also note that in practice there is little difference between using finite symmetric Dirichlet priors and stick-breaking approximations to Dirichlet processes in the context of approximate variational inference.

Therefore, if we set the number of clusters large enough, we can view the models as variants of hierarchical Dirichlet process mixtures. The number of clusters serves only as an upper bound on the number of clusters that we can expect in the data. Only a smaller number of clusters will

be required to represent the data in the posterior. We can also use the theory behind Dirichlet processes to approximate the number of clusters that we can expect in the data [4]:

$$\mathbb{E}[S] \simeq \gamma \log\left(1 + \frac{N}{\gamma}\right)$$

where we used the notation from the decoupled model (5). This result shows that the expected number of clusters grows only logarithmically with the number of data points N.

Dirichlet processes have been widely used to estimate the number of components in complex data, for example by Medvedovic et al. [5] in context of gene expression clustering. However, we should note that Dirichlet processes do not estimate the correct number of clusters in the posterior. This behaviour was both shown to be a theoretical property of the Dirichlet process by Miller and Harrison [6] and observed in practice [7].

Miller and Harrison [6] also note that this inconsistency is caused by the tendency of Dirichlet processes to allocate some number of very small clusters. Bearing this behaviour in mind, when we are interested in the posterior number of clusters we can look only at larger clusters which form a good representation of the modelled data.

Moving past simple Dirichlet processes, the two models presented in this chapter are also closely related to the hierarchical Dirichlet process (HDP, [8]). The hierarchical Dirichlet process is a Bayesian nonparametric model which can be used for joint clustering of groups of data. It assumes that each group of data is modelled using a mixture distribution with a Dirichlet process prior. The individual groups are then linked with a hierarchical model through a common top-level Dirichlet process prior.

Teh et al. 2006 [8] present two different finite models which both lead to the hierarchical Dirichlet process (HDP) in their limit when the number of clusters goes to infinity. The two models which give rise to the hierarchical process in their infinite limit are (using notation consistent with this section):

$$\begin{aligned}
\boldsymbol{\pi} \mid \alpha_0 &\sim \text{Dirichlet}(\alpha_0/K, \ldots, \alpha_0/K) \\
\boldsymbol{\rho}_j \mid \boldsymbol{\pi}, \gamma_0 &\sim \text{Dirichlet}(\gamma_0 \boldsymbol{\pi}) \\
\theta_k \mid H &\sim H \\
z_{jn} \mid \boldsymbol{\rho}_j &\sim \text{Categorical}(\boldsymbol{\rho}_j) \\
\boldsymbol{x}_{jn} \mid z_{jn}, \{\theta_k\}_{k=1}^K &\sim F\left(x_{jn} \mid \theta_{z_{jn}}\right)
\end{aligned} \tag{S11}$$

and

$$\begin{aligned}
\boldsymbol{\pi} \mid \alpha_0 &\sim \text{Dirichlet}(\alpha_0/K, \ldots, \alpha_0/K) \\
\boldsymbol{\rho} \mid \gamma_0 &\sim \text{Dirichlet}(\gamma_0/S, \ldots, \gamma_0/S) \\
k_{js} \mid \boldsymbol{\pi} &\sim \text{Categorical}(\boldsymbol{\pi}) \\
z_{jn} \mid \boldsymbol{\rho} &\sim \text{Categorical}(\boldsymbol{\rho}) \\
\theta_k \mid H &\sim H \\
\boldsymbol{x}_{jn} \mid z_{jn}, \{k_{js}\}_{s=1}^S, \{\theta_k\}_{k=1}^K &\sim F(\boldsymbol{x}_{jn} \mid \theta_{k_{jz_{jn}}})
\end{aligned} \tag{S12}$$

where j indices the group of data $j = 1, \ldots, J$. The first model uses a similar approach to the fully combinatorial model, and the second model has a similar structure as the decoupled version.

Using the two models we can look at the context-dependent clustering as a finite representation of a hierarchical Dirichlet process, where we have only a single group of data $\boldsymbol{x}_{j1}, \ldots, \boldsymbol{x}_{jN}$, $j = 1$, but multiple top-level prior distributions, one for each context. These prior mixture distributions are grouped together and form atoms of the base distribution for the lower-level

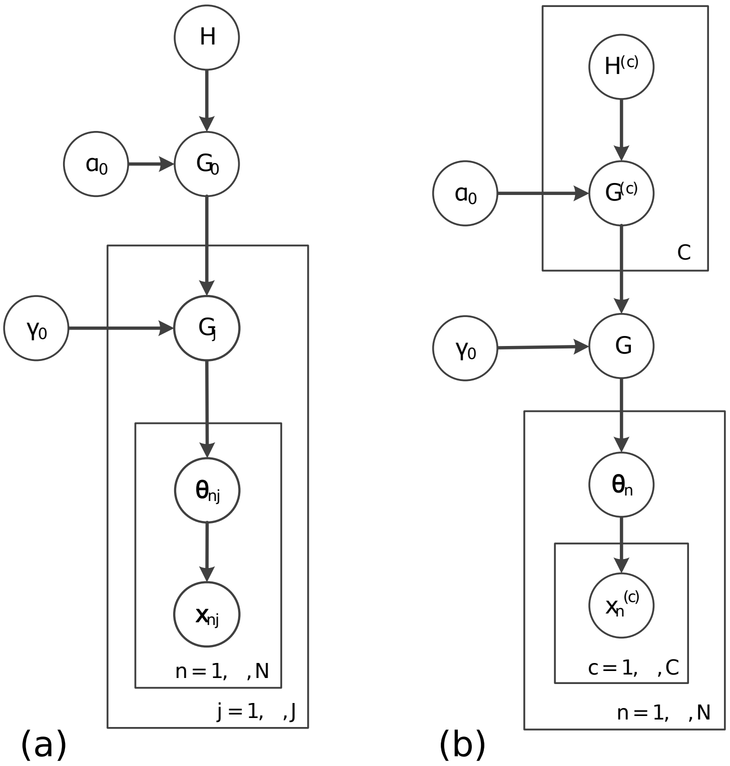

Figure 1: Graphical models of the (a) standard hierarchical Dirichlet process and (b) the limiting hierarchical Dirichlet process for the context-dependent clustering model.

Dirichlet process G. The resulting hierarchical Dirichlet process has the following form:

$$\begin{aligned}
G^{(c)} \mid \alpha_0, H^{(c)} &\sim \mathrm{DP}\left(\alpha_0, H^{(c)}\right), \qquad c = 1, \ldots, C \\
G \mid \gamma_0, G^{(1)}, \ldots, G^{(C)} &\sim \mathrm{DP}\left(\gamma_0, \bigotimes_{c=1}^{C} G^{(c)}\right) \\
\phi_n \mid G &\sim G \\
\boldsymbol{x}_n \mid \phi_n &\sim F\left(\boldsymbol{x}_n \mid \phi_n\right) = \prod_{c=1}^{C} F^{(c)}\left(\boldsymbol{x}_n^{(c)} \mid \phi_n[c]\right)
\end{aligned} \tag{S13}$$

where ϕ_n are atoms drawn from the Dirichlet process G, which are composed of tuples of atoms from context-specific Dirichlet processes $G^{(1)}, \ldots, G^{(C)}$; $\phi_n[c]$ is then the c-th element of the tuple, which represents the atom from $G^{(c)}$. Wade et al. [9] used a similar approach with coupled Dirichlet process atoms to represent a joint model for covariates and responses in the context of density estimation.

Fig. 1 compares the graphical models for standard hierarchical Dirichlet process and the Dirichlet process originating from the context-dependent clustering model.

Although both forms of the finite models (S11) and (S12) lead to the hierarchical Dirichlet process in the limit, they lead to different forms of the process. The first model (S11) leads to a formulation with stick-breaking priors, whereas the second formulation (S12) leads to the Chinese restaurant franchise process [8], which is a sampling process equivalent to the hierarchical Dirichlet process. In the original Chinese restaurant franchise metaphor, we can view the hierarchical mixture distribution as a set of restaurants (standard Dirichlet processes) which share a common set of dishes across the franchise (top-level Dirichlet process). Actual dishes served in individual restaurants may be different but they are sampled from the same top-level menu.

We can devise a similar metaphor for sampling in our context-dependent clustering: consider a Chinese restaurant where the dishes are not cooked, but instead are ordered from C caterers. Each caterer supplies only one type of dish (starter, main, side, etc.). Customers come to the restaurant and are seated at a table s with probability proportional to the number of customers already sitting at the table, or are seated at a new table with probability proportional to γ_0. Each table orders a full set of C dishes, one of each type c, with probability proportional to the number of tables that have already ordered the dish, or sample a new dish with probability proportional to α_0.

In this metaphor, customers correspond to data items $\boldsymbol{x}_1, \ldots, \boldsymbol{x}_N$ and tables represent the global clusters. Menus that are served at each table are tuples of context-specific local cluster parameters that represent the cluster in each context c.

The decoupled Clusternomics model (5) uses a similar approach as the one developed in Liu et al. [10] for clustering of two contexts: gene expression and ChIP-chip data. The paper develops a special case of the decoupled model for integration of the two specific datasets. However, our model presents a general treatment of both the local and the global clustering and the theoretical background of the model in the framework of hierarchical Dirichlet processes.

S1.2 Inference in the context-dependent clustering model

This section outlines the inference for the two models presented in the main paper. Here we derive collapsed Gibbs sampling algorithms for both models and we also look at variational inference in the fully combinatorial model.

S1.2.1 Posterior inference in the fully combinatorial Clusternomics model

First we derive distributions for posterior sampling in the fully combinatorial model (3). Because the model is non-conjugate due to a Dirichlet distribution used as a prior for the lower-level Dirichlet distribution, we use an auxiliary variable approach.

The joint distribution in the model (3) is

$$
\begin{aligned}
&p\left(\left\{\boldsymbol{\pi}^{(c)}\right\}_{c=1}^{C}, \boldsymbol{\rho}, \boldsymbol{Z}, \boldsymbol{X}^{(1)}, \ldots, \boldsymbol{X}^{(C)}, \left\{\theta_k^{(1)}, \ldots, \theta_k^{(C)}\right\}_{k=1}^{K}\right) = \\
&= \left\{\prod_{c=1}^{C} p\left(\boldsymbol{\pi}^{(c)} \mid \alpha_0\right)\right\} p\left(\boldsymbol{\rho} \mid \boldsymbol{\pi}^{(1)}, \ldots, \boldsymbol{\pi}^{(C)}, \gamma_0\right) \\
&\times \left\{\prod_{n=1}^{N} p\left(\boldsymbol{z}_n \mid \boldsymbol{\rho}\right) \left[\prod_{c=1}^{C} p\left(\boldsymbol{x}_n^{(c)} \mid \theta_{z_n[c]}^{(c)}\right)\right]\right\} \left\{\prod_{k=1}^{K}\prod_{c=1}^{C} p\left(\theta_k^{(c)} \middle| H^{(c)}\right)\right\}
\end{aligned}
$$

We perform posterior inference over the context-level mixture weights $\boldsymbol{\pi}^{(c)}$, cluster assignment variables z_n, while integrating out the global mixture weights $\boldsymbol{\rho}$ and cluster parameters $\boldsymbol{\theta}$.

We start by deriving the updates for 2 contexts to keep the notation simple and we later show how the sampling distributions look for a general number of contexts C. To integrate out ρ, we use the fact that it has a Dirichlet distribution conjugate with Categorical distribution

over cluster assignments z_n. This allows us to get an analytical solution:

$$
\begin{aligned}
p\left(\boldsymbol{Z} \mid \boldsymbol{\pi}^{(1)}, \boldsymbol{\pi}^{(2)}\right) = & \\
&= \int p\left(\boldsymbol{\rho} \mid \gamma_0, \boldsymbol{\pi}^{(1)}, \boldsymbol{\pi}^{(2)}\right) \prod_{n=1}^{N} p(z_n \mid \boldsymbol{\rho}) \ \mathrm{d}\boldsymbol{\rho} \\
&= \frac{\Gamma\left(\sum_{i=1}^{K^{(1)}} \sum_{j=1}^{K^{(2)}} \gamma_0 \pi_i^{(1)} \pi_j^{(2)}\right)}{\prod_{i=1}^{K^{(1)}} \prod_{j=1}^{K^{(2)}} \Gamma\left(\gamma_0 \pi_i^{(1)} \pi_j^{(2)}\right)} \\
&\quad \times \int \prod_{i=1}^{K^{(1)}} \prod_{j=1}^{K^{(2)}} \rho_{ij}^{\gamma_0 \pi_i^{(1)} \pi_j^{(2)} - 1} \prod_{n=1}^{N} \prod_{i=1}^{K^{(1)}} \prod_{j=1}^{K^{(2)}} \rho_{ij}^{\delta[z_n=(i,j)]} \ \mathrm{d}\boldsymbol{\rho} \\
&= \frac{\Gamma(\gamma_0)}{\prod_{i=1}^{K^{(1)}} \prod_{j=1}^{K^{(2)}} \Gamma\left(\gamma_0 \pi_i^{(1)} \pi_j^{(2)}\right)} \frac{\prod_{i=1}^{K^{(1)}} \prod_{j=1}^{K^{(2)}} \Gamma\left(\gamma_0 \pi_i^{(1)} \pi_j^{(2)} + n_{ij}\right)}{\Gamma\left(\gamma_0 + N\right)} \\
&= \frac{\Gamma(\gamma_0)}{\Gamma(\gamma_0 + N)} \prod_{i=1}^{K^{(1)}} \prod_{j=1}^{K^{(2)}} \frac{\Gamma\left(\gamma_0 \pi_i^{(1)} \pi_j^{(2)} + n_{ij}\right)}{\Gamma\left(\gamma_0 \pi_i^{(1)} \pi_j^{(2)}\right)} \qquad \text{(S14)}
\end{aligned}
$$

where $n_{ij} = \sum_{n=1}^{N} \delta[z_n = (i,j)]$.

After integrating out $\boldsymbol{\rho}$ and $\boldsymbol{\theta}$, we get the following joint distribution for general number of contexts C:

$$
\begin{aligned}
p\left(\left\{\boldsymbol{\pi}^{(c)}\right\}_{c=1}^{C}, \boldsymbol{Z}, \boldsymbol{X}^{(1)}, \ldots, \boldsymbol{X}^{(C)}\right) &= \left\{\prod_{c=1}^{C} p\left(\boldsymbol{\pi}^{(c)} \mid \alpha_0\right)\right\} \\
&\quad \times p\left(\boldsymbol{Z} \mid \boldsymbol{\pi}^{(1)}, \ldots, \boldsymbol{\pi}^{(C)}, \gamma_0\right) \prod_{c=1}^{C} \prod_{l=1}^{K^{(c)}} p\left(\boldsymbol{X}_l^{(c)} \mid H^{(c)}\right) \qquad \text{(S15)}
\end{aligned}
$$

where the individual terms are

$$
\begin{aligned}
p\left(\boldsymbol{\pi}^{(c)} \mid \alpha_0\right) &= \frac{\Gamma\left(K^{(c)} \alpha_0\right)}{\left(\Gamma(\alpha_0)\right)^{K^{(c)}}} \prod_{i=1}^{K^{(c)}} \left(\pi_i^{(c)}\right)^{\alpha_0 - 1} \\
p\left(\boldsymbol{Z} \mid \boldsymbol{\pi}^{(1)}, \ldots, \boldsymbol{\pi}^{(C)}, \gamma_0\right) & \\
&= \frac{\Gamma(\gamma_0)}{\Gamma(\gamma_0 + N)} \prod_{i_1=1}^{K^{(1)}} \cdots \prod_{i_C=1}^{K^{(C)}} \frac{\Gamma\left(\gamma_0 \pi_{i_1}^{(1)} \cdots \pi_{i_C}^{(C)} + n_{i_1,\ldots,i_C}\right)}{\Gamma\left(\gamma_0 \pi_{i_1}^{(1)} \cdots \pi_{i_C}^{(C)}\right)} \\
\boldsymbol{X}_l^{(c)} &= \left\{\boldsymbol{x}_n^{(c)}; n = 1, \ldots, N \text{ where } z_n[c] = l\right\}
\end{aligned}
$$

The data likelihood $p\left(\boldsymbol{X}_l^{(c)} \mid H^{(c)}\right)$ depends on the data distribution.

We proceed by looking at the Gibbs sampling distributions for the individual variables, using the joint distribution (S15). The posterior distribution of $\boldsymbol{z}_n$ combines the data likelihood and the derived distribution (S14), yielding

$$
\begin{aligned}
p\left(z_n = (i_1, \ldots, i_C) \mid \boldsymbol{\pi}^{(1)}, \ldots, \boldsymbol{\pi}^{(2)}, \boldsymbol{Z}^{-n}, \boldsymbol{X}\right) & \\
&\propto \left(\gamma_0 \pi_{i_1}^{(1)} \cdots \pi_{i_C}^{(C)} + n_{i_1,\ldots,i_C;-n}\right) \prod_{c=1}^{C} F\left(\boldsymbol{x}_n^{(c)} \mid \boldsymbol{X}_{i_c,-n}^{(c)}\right)
\end{aligned}
$$

where $\boldsymbol{X}^{(c)}_{i_c}$ is the set of all data points assigned to context-specific cluster i_c and the subscript $-n$ denotes that the n-th data point is excluded.

The posterior sampling distributions for $\boldsymbol{\pi}^{(c)}$ are complicated by the non-conjugacy of Dirichlet distributions. We use auxiliary variables to derive the posterior distributions, similarly to inference in hierarchical Dirichlet processes derived in Teh et al. [8]. The same inference scheme can be also derived using finite stick-breaking construction.

Again, we show the derivation using two contexts and provide the resulting equations for the general case. Starting from (S14), we introduce the auxiliary variables using the properties of the Gamma function:

$$\frac{\Gamma\left(\gamma_0 \pi_i^{(1)} \pi_j^{(2)} + n_{ij}\right)}{\Gamma\left(\gamma_0 \pi_i^{(1)} \pi_j^{(2)}\right)} = \prod_{m_{ij}=1}^{n_{ij}} \left(\gamma_0 \pi_i^{(1)} \pi_j^{(2)} + m_{ij} - 1\right)$$

which we can recognise as the rising factorial.

Coefficients of the rising factorial are the unsigned Stirling numbers of the first kind $\begin{bmatrix} n \\ k \end{bmatrix}$:

$$x(x+1)(x+2)\cdots(x+n-1) = \sum_{k=0}^{n} \begin{bmatrix} n \\ k \end{bmatrix} x^k$$

Using the unsigned Stirling numbers of the first kind, we can rewrite the equation as

$$\frac{\Gamma\left(\gamma_0 \pi_i^{(1)} \pi_j^{(2)} + n_{ij}\right)}{\Gamma\left(\gamma_0 \pi_i^{(1)} \pi_j^{(2)}\right)} = \sum_{m_{ij}=0}^{n_{ij}} \begin{bmatrix} n_{ij} \\ m_{ij} \end{bmatrix} \left(\gamma \pi_i^{(1)} \pi_j^{(2)}\right)^{m_{ij}} \tag{S16}$$

We use the variables $m_{ij}, i = 1, \ldots, K^{(1)}, j = 1, \ldots, K^{(2)}$ as auxiliary variables in our model. Putting (S16) together with the joint distribution (S15) while introducing the auxiliary variables $\boldsymbol{M} = \{m_{ij}, i = 1, \ldots, K^{(1)}, j = 1, \ldots, K^{(2)}\}$, we get the new joint distribution

$$\begin{aligned} &p\left(\boldsymbol{\pi}^{(1)}, \boldsymbol{\pi}^{(2)}, \boldsymbol{Z}, \boldsymbol{X}^{(1)}, \boldsymbol{X}^{(2)}, \boldsymbol{M}\right) = \\ &= \frac{\Gamma(K^{(1)}\alpha_0)}{\Gamma(\alpha_0)^{K^{(1)}}} \prod_{i=1}^{K^{(1)}} \left(\pi_i^{(1)}\right)^{\alpha_0 - 1} \frac{\Gamma(K^{(2)}\alpha_0)}{\Gamma(\alpha_0)^{K^{(2)}}} \prod_{j=1}^{K^{(2)}} \left(\pi_j^{(2)}\right)^{\alpha_0 - 1} \\ &\quad \frac{\Gamma(\gamma_0)}{\Gamma(\gamma_0 + N)} \prod_{i=1}^{K^{(1)}} \prod_{j=1}^{K^{(2)}} \begin{bmatrix} n_{ij} \\ m_{ij} \end{bmatrix} \left(\gamma \pi_i^{(1)} \pi_j^{(2)}\right)^{m_{ij}} \end{aligned}$$

We can see that summing out M yields the original joint distribution (S15).

Using the joint distribution with auxiliary variables, we get closed form posterior sampling distributions:

$$\begin{aligned} p\left(m_{ij} = m \mid \boldsymbol{Z}, \boldsymbol{\pi}^{(1)}, \boldsymbol{\pi}^{(2)}, \boldsymbol{M}_{-ij}\right) &\propto \begin{bmatrix} n_{ij} \\ m \end{bmatrix} \left(\gamma_0 \pi_i^{(1)} \pi_j^{(2)}\right)^m \\ p\left(\boldsymbol{\pi}^{(1)} \mid \boldsymbol{Z}, \boldsymbol{M}, \boldsymbol{\pi}^{(2)}\right) &\propto \prod_{i=1}^{K^{(1)}} \left(\pi_i^{(1)}\right)^{\alpha_0 + \sum_{j=1}^{K^{(2)}} m_{ij} - 1} \end{aligned}$$

and similarly for $\boldsymbol{\pi}^{(2)}$. Then the posterior of $\pi^{(c)}$ follows the Dirichlet distribution

$$\begin{aligned} &\boldsymbol{\pi}^{(c)} \sim \text{Dirichlet}(\boldsymbol{\alpha}^{(c)}) \\ &\alpha_k^{(c)} = \alpha_0 + m_k^{(c)} \\ &m_k^{(c)} = \sum_{i_1=1}^{K^{(1)}} \cdots \sum_{i_{c-1}}^{K^{(c-1)}} \sum_{i_{c+1}}^{K^{(c+1)}} \cdots \sum_{i_C}^{K^{(C)}} m_{i_1,\ldots,i_{c-1},k,i_{c+1},\ldots,i_C} \end{aligned}$$

where $m_k^{(c)}$ is the sum of the auxiliary variables $m_{i_1,\dots,i_C}$ over all contexts except c such that $i_c = k$. The general form of the sampling distribution for auxiliary variables $\boldsymbol{M}$ is the following:

$$p\left(m_{i_1,\dots,i_C} = m \mid \boldsymbol{Z}, \boldsymbol{\pi}^{(1)}, \dots, \boldsymbol{\pi}^{(C)}, \boldsymbol{M}_{-i_1,\dots,i_C}\right) \propto \begin{bmatrix} n_{i_1,\dots,n_C} \\ m \end{bmatrix} \left(\gamma_0 \pi_{i_1}^{(1)} \dots \pi_{i_C}^{(C)}\right)^m$$

In this section, we presented Gibbs sampling distributions for assignment variables $\boldsymbol{z}_n$ and context-specific mixture weights $\boldsymbol{\pi}^{(c)}$, integrating out the global mixture weights $\boldsymbol{\rho}$ and cluster parameters $\theta^{(c)}$. This completes the basic MCMC algorithm for sampling in the fully combinatorial Clusternomics model (3). Computational complexity of each iteration of this algorithm is $\mathcal{O}\left(NC \prod_{c=1}^{C} K^{(c)}\right)$, additionally multiplied by the complexity of evaluating the data likelihood $F^{(c)}$ for each context. The next section derives Gibbs sampling in the second form of the model - the decoupled CDC model (5).

S1.2.2 Posterior inference in the decoupled Clusternomics model

This section looks at posterior inference in the decoupled Clusternomics model in Equation (5). Gibbs sampling in this form of the model is more straightforward than the fully combinatorial because it does not use the hierarchical combination of two Dirichlet distributions, which are non-conjugate.

The joint distribution in the decoupled Clusternomics model is

$$\begin{aligned} & p\left(\boldsymbol{\rho}, \boldsymbol{z}, \boldsymbol{\pi}, \boldsymbol{k}, \boldsymbol{\theta}, \boldsymbol{X} \mid \gamma_0, \alpha_0, \boldsymbol{H}, \boldsymbol{K}, S\right) = \\ & = p\left(\boldsymbol{\rho} \mid \gamma_0\right) \times \prod_{n=1}^{N} \prod_{s=1}^{S} p(z_n = s \mid \boldsymbol{\rho}) \times \prod_{c=1}^{C} p\left(\boldsymbol{\pi}^{(c)} \mid \alpha_0\right) \times \\ & \quad \times \prod_{c=1}^{C} \left\{ \prod_{s=1}^{S} \prod_{l=1}^{K^{(c)}} p\left(k_s^{(c)} = l \mid \boldsymbol{\pi}^{(c)}\right) \times \prod_{l=1}^{K^{(c)}} p\left(\theta_l^{(c)} \mid \boldsymbol{H}^{(c)}\right) \right\} \times \\ & \quad \times \prod_{c=1}^{C} \prod_{n=1}^{N} p\left(x_n^{(c)} \mid \theta_{k_{z_n}}^{(c)}\right) \end{aligned}$$

As in the fully combinatorial model, we can integrate out some of the variables: both the global mixture weights $\boldsymbol{\rho}$ and context-specific mixture weights $\boldsymbol{\pi}^{(c)}$, as well as the cluster parameters $\boldsymbol{\theta}^{(c)}$.

To marginalise the mixture weights $\boldsymbol{\rho}$ and $\boldsymbol{\pi}^{(c)}$, we again use the conjugacy of the Dirichlet distribution with the Categorical:

$$\begin{aligned} p(\boldsymbol{z} \mid \gamma_0) &= \int p(\boldsymbol{\rho} \mid \gamma_0) \prod_{n=1}^{N} p(z_n \mid \boldsymbol{\rho}) \, \mathrm{d}\boldsymbol{\rho} \\ &= \frac{\Gamma(\gamma_0)}{\Gamma\left(\frac{\gamma_0}{S}\right)^S} \int \prod_{s=1}^{S} \rho_s^{\gamma_0/S + n_s - 1} \, \mathrm{d}\boldsymbol{\rho} \\ &= \frac{\Gamma(\gamma_0)}{\Gamma\left(\frac{\gamma_0}{S}\right)^S} \times \frac{\prod_{s=1}^{S} \Gamma\left(\frac{\gamma_0}{S} + n_s\right)}{\Gamma(\gamma_0 + N)} \end{aligned}$$

where n_s is the number of data points assigned to global cluster $s : n_s = \sum_{n=1}^{N} \delta[z_n = s]$.

We can use this expression to derive conditional probability of z_n given all other assignments, which we need in the Gibbs sampler:

$$p\left(z_n = s \mid \boldsymbol{Z}_{-n}, \gamma_0\right) = \frac{p\left(z_n, \boldsymbol{Z}_{-n} \mid \gamma_0\right)}{p\left(\boldsymbol{Z}_{-n} \mid \gamma_0\right)} = \frac{\frac{\gamma_0}{S} + n_{s\cdot,-n}^{(\cdot)}}{\gamma_0 + N - 1}$$

where $n_{s,-n} = \sum_{n' \neq n} \delta[z_{n'} = s]$ is the number of data points assigned to cluster s, leaving out the n-th data point.

Similarly, we can integrate out the context-specific mixture weights $\boldsymbol{\pi}^{(c)}$ and get the following expressions:

$$p\left(\boldsymbol{k}^{(c)} \mid \alpha_0\right) = \frac{\Gamma(\alpha_0)}{\Gamma\left(\frac{\alpha_0}{K^{(c)}}\right)^{K^{(c)}}} \times \frac{\prod_{l=1}^{K^{(c)}} \Gamma\left(\frac{\alpha_0}{K^{(c)}} + m_l^{(c)}\right)}{\Gamma\left(\alpha_0 + K^{(c)}\right)}$$

$$p\left(k_s^{(c)} = l \mid \boldsymbol{k}_{-s}^{(c)}, \alpha_0\right) = \frac{\frac{\alpha_0}{K^{(c)}} + m_{l,-s}^{(c)}}{\alpha_0 + S - 1}$$

where $m_l^{(c)}$ is number of global clusters that map to context-cluster l in context c: $m_l^{(c)} = \sum_{s=1}^{S} \delta\left[k_s^{(c)} = l\right]$. The additional subscript $-s$ denotes omission of the s-th global cluster. Looking at m_l and using the hierarchical Dirichlet process metaphor, $m_l^{(c)}$ would correspond to the number of tables serving dish l, which is the c-th item in the menu.

If the data distribution $F^{(c)}$ is conjugate with respect to $H^{(c)}$, we can integrate out the cluster parameters $\boldsymbol{\theta}^{(c)}$ as well, giving us the following Gibbs sampling distributions for z_n and $k_s^{(c)}$:

$$p(z_n = s \mid \boldsymbol{z}_{-n}, \boldsymbol{X}, \gamma_0) \propto \frac{\frac{\gamma_0}{S} + n_{s,-n}}{\gamma_0 + N - 1} \prod_{c=1}^{C} F^{(c)}\left(\boldsymbol{x}_n^{(c)} \mid \boldsymbol{X}_{l,-n}^{(c)}, \boldsymbol{H}^{(c)}\right) \tag{S17}$$

and

$$p\left(k_s^{(c)} = l \mid \boldsymbol{k}_{-s}^{(c)}, \alpha_0, \boldsymbol{X}\right) \propto \frac{\frac{\alpha_0}{K^{(c)}} + m_{l,-s}^{(c)}}{\alpha_0 + S - 1} \times F^{(c)}\left(\boldsymbol{X}_s^{(c)} \mid \boldsymbol{X}_{l,-s}^{(c)}, \boldsymbol{H}^{(c)}\right) \tag{S18}$$

where the subscripts $-n$ and $-s$ represent the omission of the n-th data point and s-th global cluster respectively, and

$$\boldsymbol{X}_s^{(c)} = \left\{x_n^{(c)}; n = 1, \ldots, N \text{ where } z_n = s\right\}$$

is the set of data points in context c assigned to global cluster s.

Finally, after we marginalise out the mixture weights $\boldsymbol{\rho}$ and $\boldsymbol{\pi}^{(c)}$, and cluster parameters $\boldsymbol{\theta}^{(c)}$, we get the following joint likelihood:

$$p(\boldsymbol{z}, \boldsymbol{k}, \boldsymbol{X} \mid \gamma_0, \alpha_0, \boldsymbol{H}, \boldsymbol{K}, S) = p(\boldsymbol{z} \mid \gamma_0) \prod_{c=1}^{C} p\left(\boldsymbol{k}^{(c)} \mid \alpha_0\right) \prod_{c=1}^{C} \prod_{l=1}^{K^{(c)}} p\left(\boldsymbol{X}_l^{(c)} \mid \boldsymbol{H}^{(c)}\right) \tag{S19}$$

where the individual terms are:

$$\boldsymbol{X}_l^{(c)} = \left\{x_n^{(c)}; n = 1, \ldots, N \text{ where } k_{z_n} = l\right\} \tag{S110}$$

$$p(\boldsymbol{z} \mid \gamma_0) = \frac{\Gamma(\gamma_0)}{\Gamma\left(\frac{\gamma_0}{S}\right)^S} \times \frac{\prod_{s=1}^{S} \Gamma\left(\frac{\gamma_0}{S} + n_s\right)}{\Gamma(\gamma_0 + N)} \tag{S111}$$

$$p\left(\boldsymbol{k}^{(c)} \mid \alpha_0\right) = \frac{\Gamma(\alpha_0)}{\Gamma\left(\frac{\alpha_0}{K^{(c)}}\right)^{K^{(c)}}} \times \frac{\prod_{l=1}^{K^{(c)}} \Gamma\left(\frac{\alpha_0}{K^{(c)}} + m_l^{(c)}\right)}{\Gamma(\alpha_0 + S)} \tag{S112}$$

This completes the derivation of Gibbs sampling distributions for the decoupled Clusternomics model. Computational complexity of each iteration of the algorithm is $\mathcal{O}\left(NCS + S\sum_{c=1}^{C} K^{(c)}\right)$, additionally increased by the complexity of evaluating data likelihood $F^{(c)}$. Compared to the fully combinatorial model, complexity of this algorithm is lower for $S < \prod_{c=1}^{C} K^{(c)}$ because of the decoupled representation.

S1.2.3 Variational inference in the fully combinatorial model

Although collapsed Gibbs sampling is unbiased, the MCMC chain may take a long time to converge in larger and more complex datasets. The inference is inefficient because every Gibbs sampling iteration requires sampling of assignments for every individual data point. Also, to obtain estimates of the posterior distribution which is also necessary to summarise the MCMC samples.

Variational inference represents a popular alternative to Gibbs sampling because it is significantly faster than collapsed Gibbs sampling, making it more suitable for application to large real-world datasets. In this section we briefly look at mean-field variational inference and we present a simple approximate variational algorithm for the Clusternomics model with fully combinatorial structure (3).

Variational inference in general is a deterministic algorithm for approximate inference in probabilistic models [11]. Suppose we want to estimate the true probability distribution p^* which is intractable. We can approximate it by using a simpler distribution q which is tractable and easier to work with. We make the approximate distribution q as close as possible to p^* by minimising the reverse Kullback-Leibler distance between the distributions:

$$\mathbb{KL}\left(q||p^*\right) = \int q(\boldsymbol{Y}) \log \frac{q(\boldsymbol{Y})}{p^*(\boldsymbol{Y})} \,\mathrm{d}\boldsymbol{Y}$$

where $\boldsymbol{Y}$ is the set of all unknown variables in the model. This formulation also transforms inference into an optimisation problem. In general, p^* is substituted with its un-normalised form, $\tilde{p}$,

$$\tilde{p}(\boldsymbol{Y}) = p(\boldsymbol{Y}, \boldsymbol{X}) = p^*(\boldsymbol{Y}) p(\boldsymbol{X})$$

where $\boldsymbol{X}$ represents observed data and $p(\boldsymbol{X})$ serves as the normalising constant. Following the derivations given by Murphy [12], then

$$\mathbb{KL}\left(q||p^*\right) = \mathbb{KL}\left(q||\tilde{p}\right) - \log p(\boldsymbol{X})$$

The task of minimising the KL divergence can then be also expressed as the problem of maximising the lower bound on data log likelihood:

$$\mathcal{L}(q) \mathrel{\hat{=}} -KL\left(q||\tilde{p}\right) + \log p(\boldsymbol{X}) \leq \log p(\boldsymbol{X})$$

Here $\mathcal{L}$ is the so-called variational lower bound. The value of the variational lower bound can be used to assess the convergence of the algorithm and it is also invaluable during debugging, because its value should never decrease during optimisation.

A popular choice for the distribution q is the mean field approximation [13]: a fully factorised distribution over all latent variables and parameters $\boldsymbol{Y}$:

$$q(\boldsymbol{Y}) = \prod_i q_i(Y_i)$$

This factorisation breaks dependencies that exist between the latent variables and parameters, making inference in complex models tractable. For the Clusternomics model, the mean-field form of the function q is the following:

$$q\left(\pi^{(1)}, \ldots, \pi^{(C)}, \boldsymbol{\rho}, \boldsymbol{Z}, \boldsymbol{\theta}^{(1)}, \ldots, \boldsymbol{\theta}^{(C)}\right) = q(\boldsymbol{\rho})\; q(\boldsymbol{Z}) \prod_{c=1}^{C} q(\pi^{(c)})\; q(\boldsymbol{\theta}^{(c)})$$

In the mean field method, optimising the KL divergence with respect to each unknown variable leads to coordinate descent algorithm. Each step is given by solution of the equation (for derivation of the result see for example [12]):

$$\log q_i(Y_i) = \left\langle \log \tilde{p}(\boldsymbol{Y}) \right\rangle_{q_{-i}} + \text{const}$$

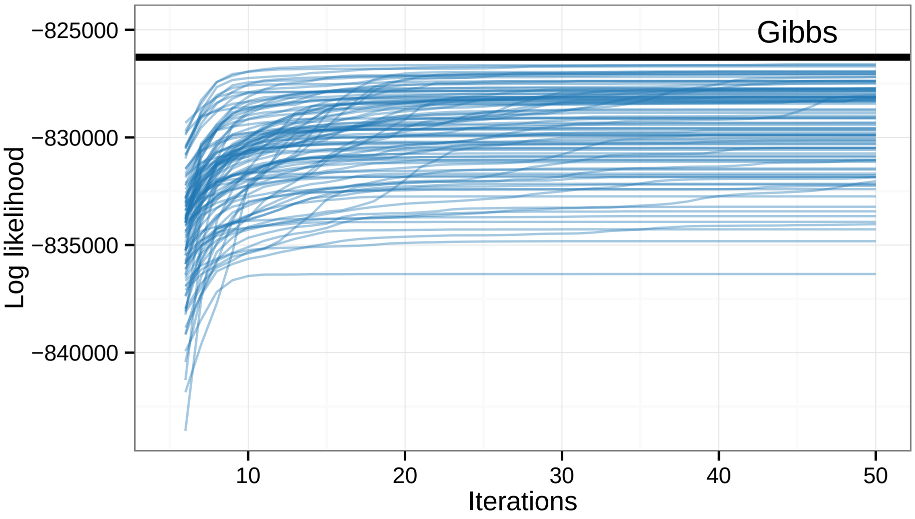

Figure 2: Lower bound on log likelihood computed for 100 runs of the variational inference algorithm described in Section 2.3, compared with the average log likelihood obtained from Gibbs sampling on the same model. Most of the runs converge within 50 iterations.

where $\langle\cdot\rangle_q = \mathbb{E}_q[\cdot]$ is the expectation with respect to distribution q, and $q_{-i} = \prod_{i\neq j} q_j$ are all the mean field distributions except q_i. Therefore, each variational distribution here is updated using the mean values of other variational distributions.

Here we derive only a simplified version of the algorithm, combining variational inference with an EM-style approach. The full variational inference is applied only to the global mixture weights $\boldsymbol{\rho}$, assignment variables $\boldsymbol{z}$ and cluster parameters $\boldsymbol{\theta}$. We use standard optimisation to update values of the context-specific mixture weights $\boldsymbol{\pi}^{(c)}$. It is also possible to derive a fully variational algorithm, for example using approaches similar to the ones applied to hierarchical Dirichlet processes in Teh et al. [14] or Hughes et al. [15].

The presented algorithm gives us an efficient method to estimate clustering in the Clusternomics model. One of the main limitations of variational inference is that it can yield biased results [16], especially in cases where there is only a small number of data points assigned to some of the mixture components [17]. Unfortunately, this is often the case for currently available biological and genomic datasets, where the data points represent patients. When applying variational inference to real-world data for cancer subtyping, we found that variational inference is prone to getting trapped in local optima and many re-runs are necessary to obtain a good result. However, we can use variational inference as a fast way to initialise MCMC chain for collapsed Gibbs sampling, giving faster convergence.

In the main paper we use the collapsed Gibbs sampling to evaluate the model because it was shown to have better test set performance than models fitted with variational inference [14, 18]. Nevertheless, the efficiency of variational inference makes it a very popular algorithm for fitting Dirichlet process-type of models making it applicable to large datasets.

Figure 2 shows the variational lower bound for iterations 5 to 50 for 100 runs of the variational inference algorithm, compared to the mean posterior log likelihood of an equivalent model obtained with the Gibbs sampling. Although the variational inference suffers from local maxima in the log likelihood, it converges very quickly. The runtime for the plotted 100 runs of variational inference was also lower than for the single Gibbs sampling chain.

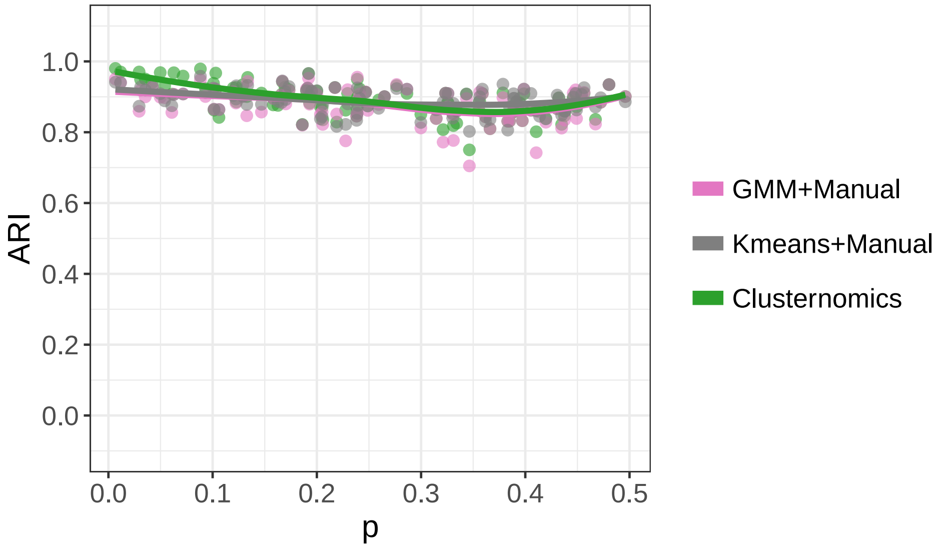

Figure 3: Comparison of Clusternomics with independent clustering within each context (dataset) followed by manual construction of global clusters, applied to simulated datasets described in the main paper. The plot shows the ARI values comparing results of the algorithms where higher values correspond to better agreement between the estimated cluster assignments and the true cluster membership. The comparison shows two types of manual integration: with k-means clustering in each dataset (Kmeans+Manual), and using Gaussian mixture model clustering (GMM+Manual). All methods have similar performance on this dataset.

S1.3 Implementation and results

Here we provide some additional technical details on the context-dependent clustering algorithm and its implementation. The decoupled Clusternomics algorithm was used on the datasets presented in the main paper. Implementation of the algorithm in R is available from CRAN as the `clusternomics` package.

S1.3.1 Clusternomics and ad-hoc integrative clustering

In this section we compare the Clusternomics method with an ad-hoc integration of local clustering assignments. The *ad-hoc* manual integrative clustering works as a two-step procedure:

1. Cluster data within each data source (context) independently, to get local cluster assignments.
2. Use combinations of local cluster assignments to manually construct global clusters.

This form of integrative clustering does not share any information between contexts.

For the small simulated dataset presented in the main paper, the results we get when using the ad-hoc integrative approach are equivalent because the small dataset has only well-defined clusters. The example illustrates mainly how existing approaches deal with situations where the cluster structure is not consistent across the different contexts. Figure 3 shows the ARI values for the range of simulated datasets, comparing Clusternomics and two versions of ad-hoc clustering: using k-means and Gaussian mixture models for local clustering. The results of all three algorithms are equivalent for the simulated dataset.

The difference between Clusternomics and manual integration of independent local clusters appears when applied to a real-world dataset where sharing of information between datasets is beneficial. Clusternomics encourages clusters to be shared across contexts: if two data points

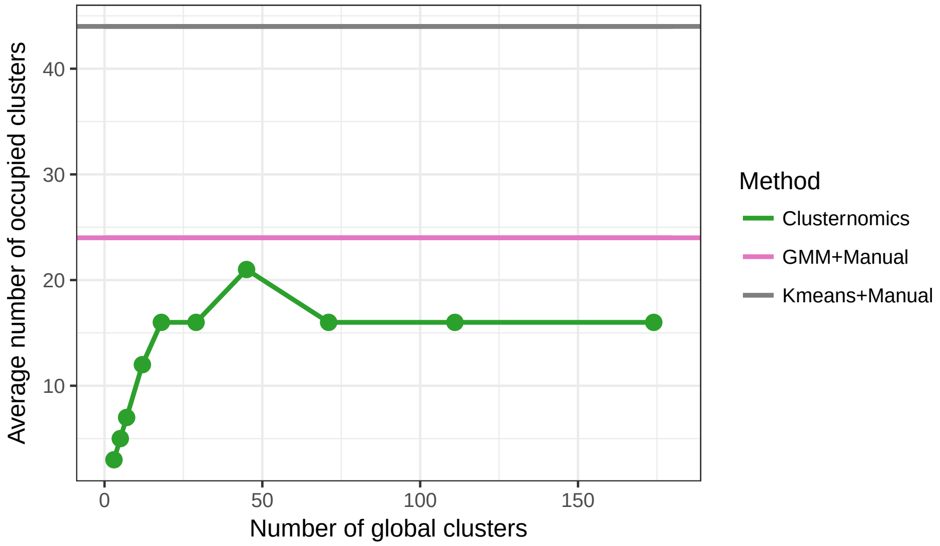

Figure 4: Comparison of Clusternomics with independent clustering within each context (dataset) followed by manual construction of global clusters, applied to the breast cancer dataset. The comparison shows the number of global clusters estimated by Clusternomics, and constructed using the two local clustering methods: k-means (Kmeans+Manual), and Gaussian mixture model with diagonal covariance matrix (GMM+Manual).

are clustered together in one dataset, they have a higher posterior probability of being clustered together in other contexts as well.

We applied the two variants of ad-hoc clustering to the breast cancer dataset from TCGA described in the main paper. Figure 4 shows the number of global clusters constructed from local cluster assignments in the breast cancer dataset, using three local clusters per context. We used the Gaussian mixture model with diagonal covariance matrix implemented in the R package `mclust` [19], and standard k-means algorithm implemented in the R function `kmeans`.

The ad-hoc integration using k-means algorithm shows the largest number of estimated global clusters: 44. The Gaussian mixture model-based integration, which is methodically closer to Clusternomics, identified 24 global clusters. The Clusternomics algorithm identified 16 global clusters (depending on the setting of the parameter). The global clusters identified by manually integrating local clusters is larger than the number of clusters estimated by Clusternomics, because the ad-hoc integration fails to share information between contexts.

The ad-hoc integration also produces a large number of small clusters. The number of global clusters with 5 or fewer data points was 28 for the k-means based clustering, and 7 for the GMM-based clustering, compared to 2 clusters in Clusternomics (see Fig. 14 in the main paper). The large number of small clusters makes the results fragmented and less interpretable.

We also looked at the resulting partitioning of the data and how it relates to the survival probabilities of individual clusters. For the k-means based clustering, the resulting clusters were not significant based on their survival probabilities (p-value 0.834). For the Gaussian mixture model based clustering, the clusters reached statistical significance (p-value ¡ 0.05). However, as we argued before, the large number of fragmented clusters identified by this method is less interpretable.

S1.3.2 MCMC sampling and runtime

Here we look at the convergence of the MCMC sampling algorithm on the TCGA datasets. We ran the Gibbs sampling algorithm for 10,000 iterations. We discarded the first 5,000 iterations as burn-in, and used every third sample. We repeated this procedure for different numbers of

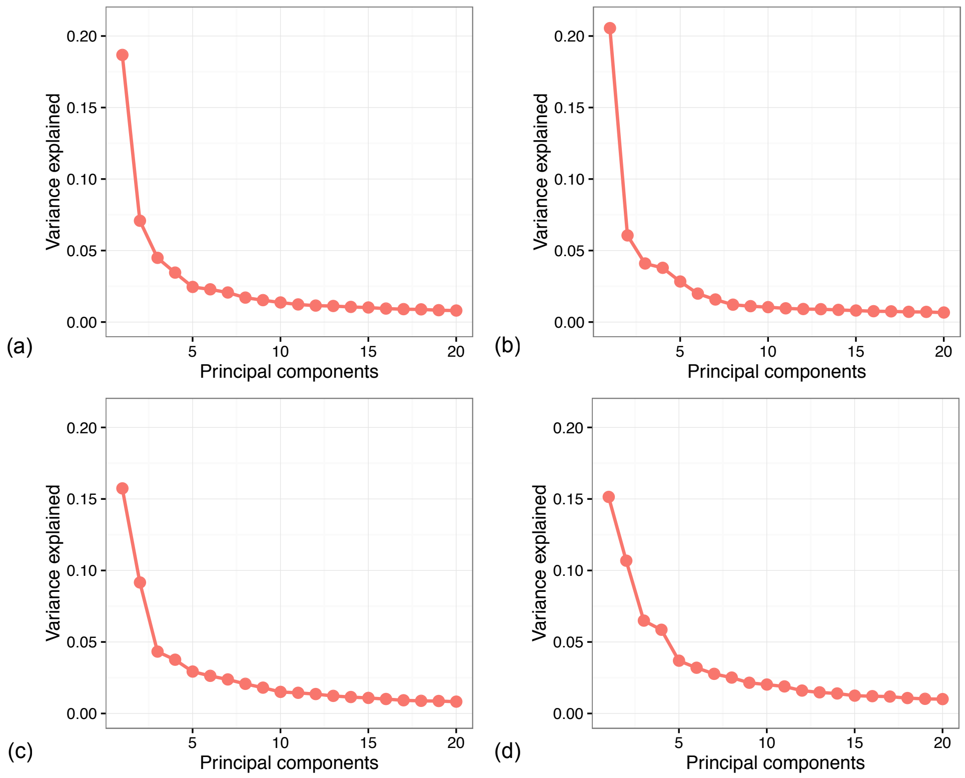

Figure 5: This plot shows the proportion of variance explained by the first twenty principal components for the breast cancer TCGA dataset. The individual figures represent a) gene expression dataset (b) DNA methylation dataset (c) miRNA expression dataset (d) RPPA dataset.

global clusters to assess the stability of the algorithm. Each full chain took approximately 3 hours to run on an Intel Xeon 2.60GHz CPU on the breast cancer dataset, 12 hours on the kidney dataset and 16 hours on the lung dataset. Longer runtime on the kidney and lung datasets was caused by their high dimensionality and our setting of larger number of global clusters.

S1.3.3 Parameter settings

S1.3.3.1 Simulated datasets

The settings of the algorithms compared in the main text were the following:

Clusternomics The number of local context-specific clusters was set to two in each context, and four globally. Concentration parameters α_0 and γ_0 were given $\text{Gamma}(1, 1)$ prior distribution. The number of iterations was 10,000.

BCC We set the number of clusters to 4 overall, running the algorithm for 10,000 iterations. The adherence parameter α was given $\text{Beta}(1, 1)$ prior distribution and it was fitted separately in each context.

iCluster The algorithm was run with $k = 4$ clusters for up to 10,000 iterations. Other parameters were left at their default values.

MDI The algorithm was run with default parameters for 10,000 iterations, assuming Gaussian distribution of the data.

SNF The algorithm was run using the R package SNFtool with default parameters, using pairwise Euclidean distance and number of clusters $C = 4$.

S1.3.3.2 Cancer subtyping

To model the three cancer datasets presented in the main paper, we used a multivariate Gaussian distribution with a diagonal covariance matrix for all the data contexts. We ran the Gibbs sampling algorithm for 10,000 iterations. We discarded the first 5,000 iterations as burn-in, and used every third sample. We repeated this procedure for different numbers of global clusters to assess the stability of the algorithm. Each full chain took approximately 3 hours to run on an Intel Xeon 2.60GHz CPU on the breast cancer dataset, 12 hours on the kidney dataset and 16 hours on the lung dataset. Longer runtime on the kidney and lung datasets was caused by their high dimensionality and our setting of larger number of global clusters.

We summarised the results after burn-in using the method by Dahl [20] using the posterior co-clustering matrix to infer the posterior cluster assignments. The model is not very sensitive to the value of the concentration parameters α_0 and γ_0. In the inference we used the following small fixed values to encourage smaller number of clusters: $\alpha_0 = 0.1$ and $\gamma_0 = 0.001$.

S1.3.4 Stability of results: posterior co-clustering matrices

To assess convergence of the MCMC algorithm, we ran 10 parallel chains and compared the co-clustering matrices resulting from samples from each of the chains. The posterior co-clustering matrix computes the posterior probability of data points being assigned to the same component. The value in row i and column j is the posterior probability of data points i and j being assigned to the same global cluster, estimated from the MCMC samples. If the parallel MCMC chains converged to the same clustering solution, they should result in similar co-clustering matrices.

Figures 6 and 7 show the co-clustering matrices from the 10 parallel MCMC chains from the breast cancer TCGA dataset. The number of context-specific clusters $K^{(c)} = 3$, and the number of global clusters $S = 18$. Figure 6 shows the co-clustering matrix of the first MCMC chain as a heatmap. Heatmaps corresponding to the other chains are shown in Figure 7. The ordering of rows and columns in the co-clustering matrices is the same as in the visualization of chain 1 to facilitate comparison. Overall, there is a good agreement between the individual chains, especially for the main clusters.

Figures 8 and 9 show similar comparison for the lung cancer TCGA dataset. The number of context-specific clusters $K^{(c)}$ was set to 3, and the number of global clusters S to 20. For this dataset, the results are again relatively stable across the individual MCMC chains.

Finally, figures 10 and 11 show the posterior co-clustering comparison for the kidney cancer TCGA dataset. The number of context-specific clusters $K^{(c)}$ was set to 3, and the number of global clusters S to 31. In this case all the individual chains converge to a very similar co-clustering matrix.

S1.3.5 Choosing number of clusters

We investigated some commonly used criteria for cluster consistency, namely the Dunn index [21] and the average Silhouette score [22]. Both measures were used to select optimal numbers of clusters in various settings, for example Curtis et al. [23] estimate the number of clusters in integrative analysis using iCluster on the METABRIC dataset using the Dunn index. The Silhouette score was used for example in Wang et al. [24] to evaluate consistency of integrative clusters.

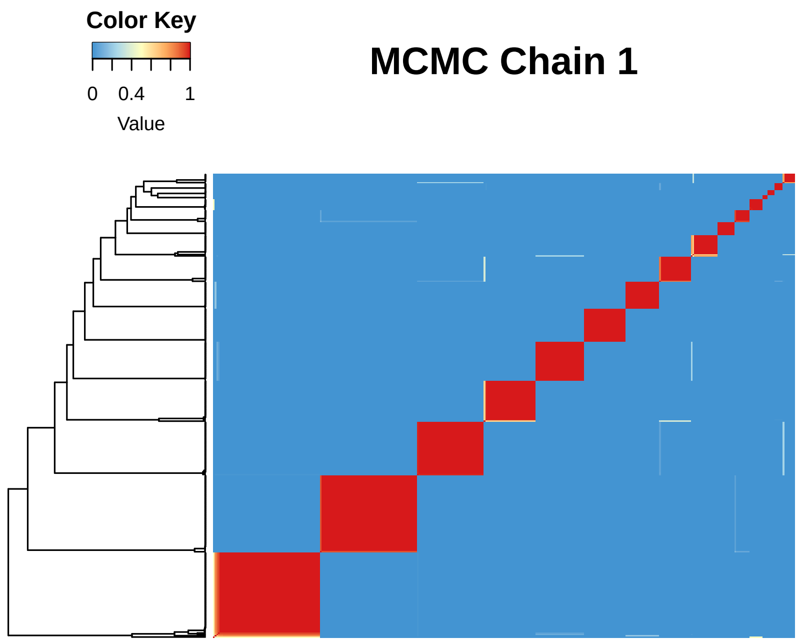

Figure 6: Heatmap visualization of the posterior co-clustering matrix of Gibbs sampling MCMC chain number 1 from the TCGA breast cancer example, where $K^{(c)} = 3$ and $S = 18$.. The value in row i and column j is the estimated posterior probability of samples i and j being assigned to the same global cluster.

Fig. 12 shows values of these measures for the individual results. Neither of the criteria is maximised for the models with good separation of survival prospects. Such criteria therefore seem less suitable for this type of high-dimensional data.

References

[1] Ishwaran H, Zarepour M. Markov chain Monte Carlo in approximate Dirichlet and beta two-parameter process hierarchical models. Biometrika. 2000;87(2):371–390.

[2] Ishwaran H, Zarepour M. Exact and approximate sum representations for the Dirichlet process. The Canadian Journal of Statistics. 2002;30(2):269–283.

[3] Kurihara K, Welling M, Teh YW. Collapsed variational Dirichlet process mixture models. International Joint Conference on Artificial Intelligence. 2007;.

[4] Teh YW. Dirichlet process. In: Encyclopedia of Machine Learning. Springer; 2010. .

[5] Medvedovic M, Sivaganesan S. Bayesian infinite mixture model based clustering of gene expression profiles. Bioinformatics. 2002 Sep;18(9):1194–1206.

[6] Miller JW, Harrison MT. A simple example of Dirichlet process mixture inconsistency for the number of components. Advances in Neural Information Processing Systems (NIPS). 2013;26.

[7] West M, Müller PM, Escobar MD. Hierarchical priors and mixture models, with application in regression and density estimation. Institute of Statistics and Decision Sciences, Duke University; 1993.

[8] Teh YW, Jordan MI, Beal MJ, Blei DM. Hierarchical Dirichlet processes; 2006. 476.

[9] Wade S, Dunson DB, Petrone S, Trippa L. Improving Prediction from Dirichlet Process Mixtures via Enrichment. Journal of Machine Learning Research. 2014;15:1041–1071.

[10] Liu X, Jessen WJ, Sivaganesan S, Aronow BJ, Medvedovic M. Bayesian hierarchical model for transcriptional module discovery by jointly modeling gene expression and ChIP-chip data. BMC Bioinformatics. 2007;8(283).

[11] Jordan MI, Ghahramani Z, Jaakkola TS, Saul LK. Introduction to variational methods for graphical models. Machine Learning. 1999;37(2):183–233.

[12] Murphy K. Machine learning: a probabilistic perspective. MIT Press; 2012.

[13] Opper M, Saad D. Advanced Mean Field Methods: Theory and Practice; 2001.

[14] Teh YW, Kurihara K, Welling M. Collapsed variational inference for HDP. Advances in Neural Information Processing Systems. 2008;20(20):1481–1488.

[15] Hughes MC, Kim DI, Sudderth EB. Reliable and Scalable Variational Inference for the Hierarchical Dirichlet Process. In: Artificial Intelligence & Statistics; 2015. p. 370–378.

[16] Buntine W, Jakulin A. Applying Discrete PCA in Data Analysis. Proceedings of the Twentieth Conference on Uncertainty in Artificial Intelligence (UAI2004). 2004;p. 59–66.

[17] Welling M, Teh YW, Kappen B. Hybrid Variational/Gibbs Collapsed Inference in Topic Models. In: Proceedings of the Twenty-Fourth Conference on Uncertainty in Artificial Intelligence (UAI2008); 2008. .

[18] Buntine W, Mishra S. Experiments with Nonn-parametric Topic Models. In: Proceedings of the 20th ACM SIGKDD International Conference on Knowledge Discovery and Data Mining. vol. KDD '14; 2014. p. 881–890.

[19] Fraley C, Raftery AE, Murphy TB, Scrucca L. mclust Version 4 for R: Normal Mixture Modeling for Model-Based Clustering, Classification, and Density Estimation. Department of Statistics, University of Washington; 2012. 597.

[20] Dahl DB. Model-based clustering for expression data via a Dirichlet process mixture model. In: Vannucci M, Do KA, Müller P, editors. Bayesian inference for gene expression and Proteomics. Cambridge University Press; 2006. .

[21] Dunn JC. A Fuzzy Relative of the ISODATA Process and Its Use in Detecting Compact Well-Separated Clusters. Journal of Cybernetics. 1973;3(3):32–57.

[22] Rousseeuw PJ. Silhouettes: A graphical aid to the interpretation and validation of cluster analysis. Journal of Computational and Applied Mathematics. 1987;20:53–65.

[23] Curtis C, Shah SP, Chin SF, Turashvili G, Rueda OM, Dunning MJ, et al. The genomic and transcriptomic architecture of 2,000 breast tumours reveals novel subgroups. Nature. 2012 Jun;486(7403):346–52.

[24] Wang B, Mezlini AM, Demir F, Fiume M, Tu Z, Brudno M, et al. Similarity network fusion for aggregating data types on a genomic scale. Nature methods. 2014;11(3):333–7.

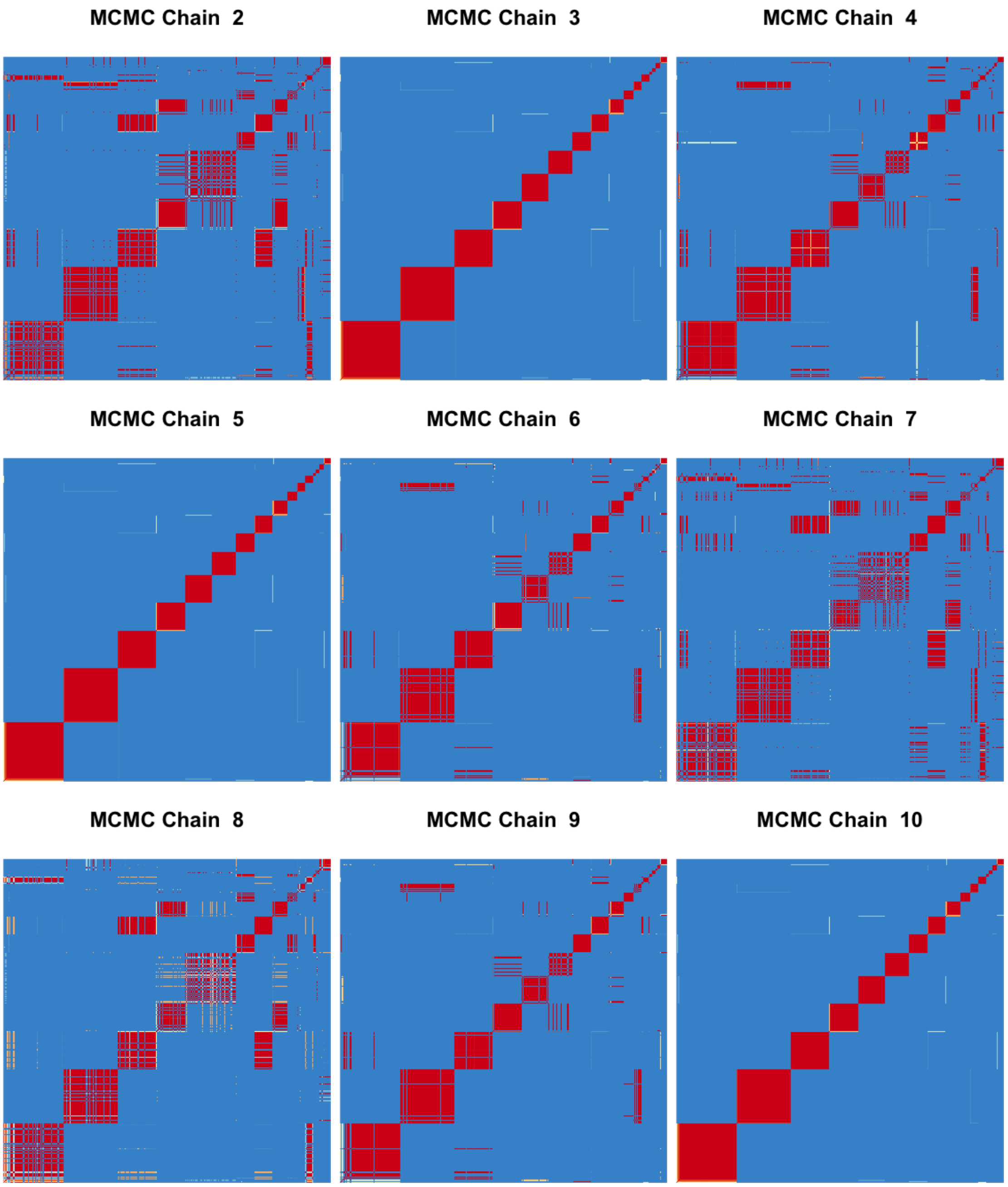

Figure 7: Heatmap visualizations of the posterior co-clustering matrices of Gibbs sampling MCMC chains number 2 to 10 from the TCGA breast cancer example. The rows and columns have the same order as in Figure 6 to facilitate comparison. The number of context-specific clusters $K^{(c)} = 3$, and the number of global clusters $S = 18$.

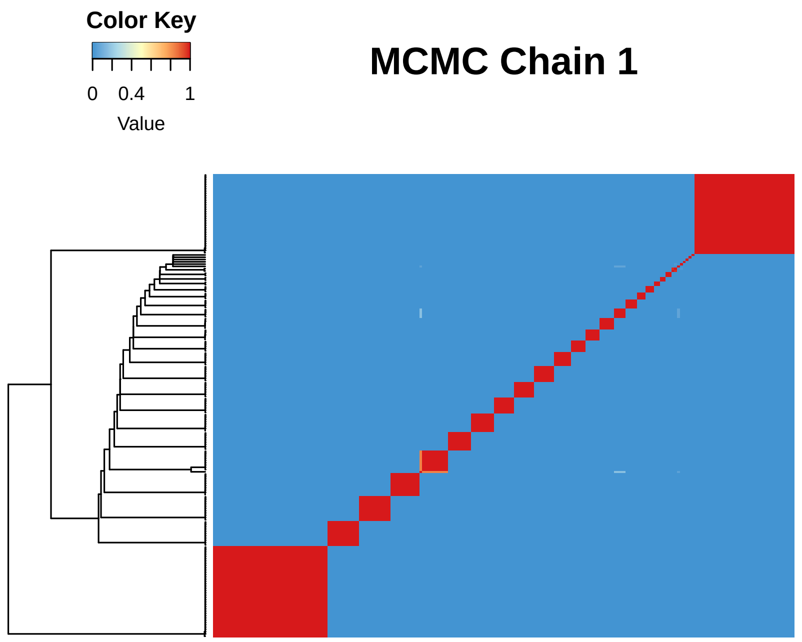

Figure 8: Heatmap visualization of the posterior co-clustering matrix of Gibbs sampling MCMC chain number 1 from the TCGA lung cancer example. The value in row i and column j is the estimated posterior probability of samples i and j being assigned to the same global cluster.

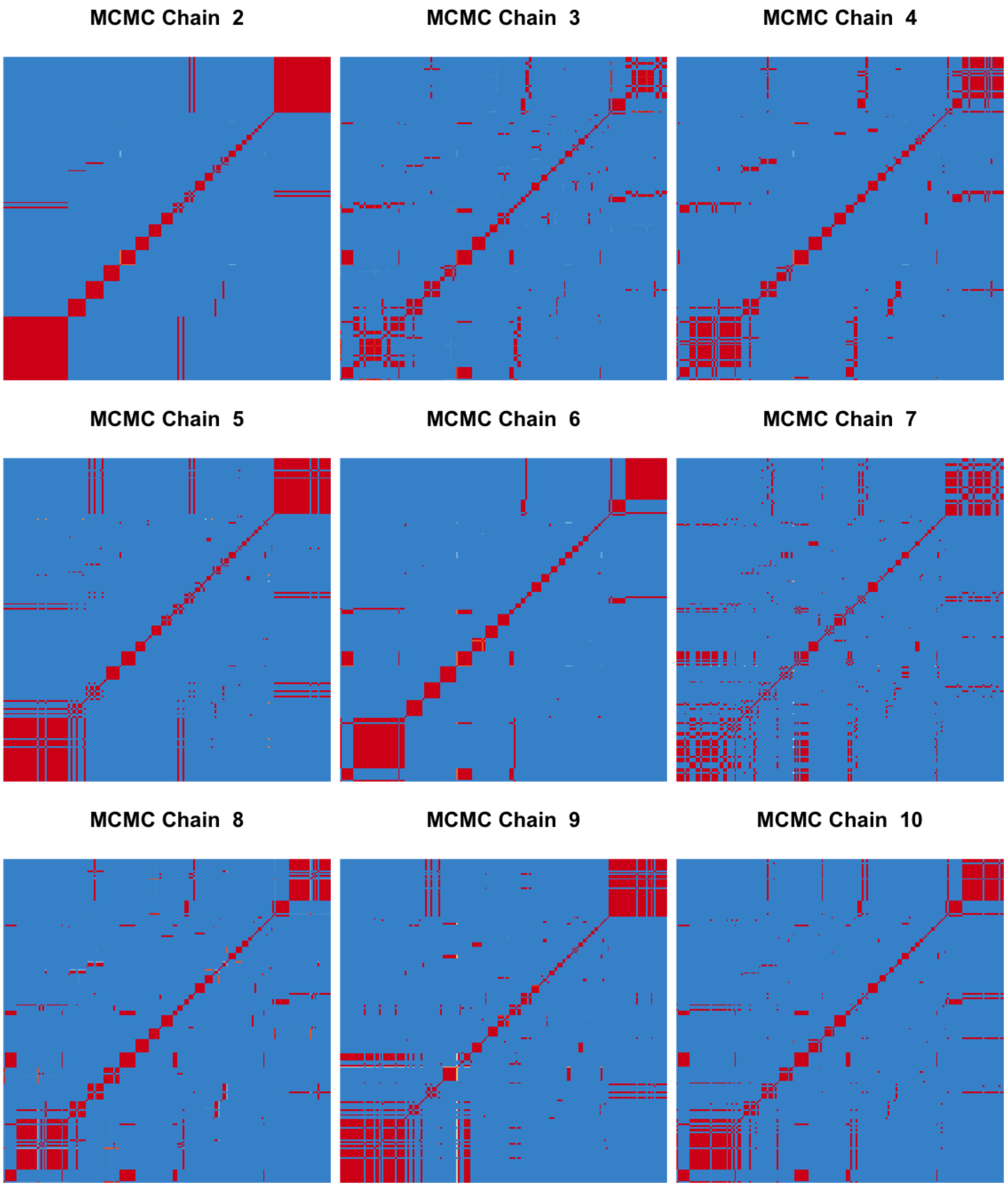

Figure 9: Heatmap visualizations of the posterior co-clustering matrices of Gibbs sampling MCMC chains number 2 to 10 from the TCGA lung cancer example. The rows and columns have the same order as in Figure 8 to facilitate comparison. The number of context-specific clusters $K^{(c)} = 3$, and the number of global clusters $S = 20$.

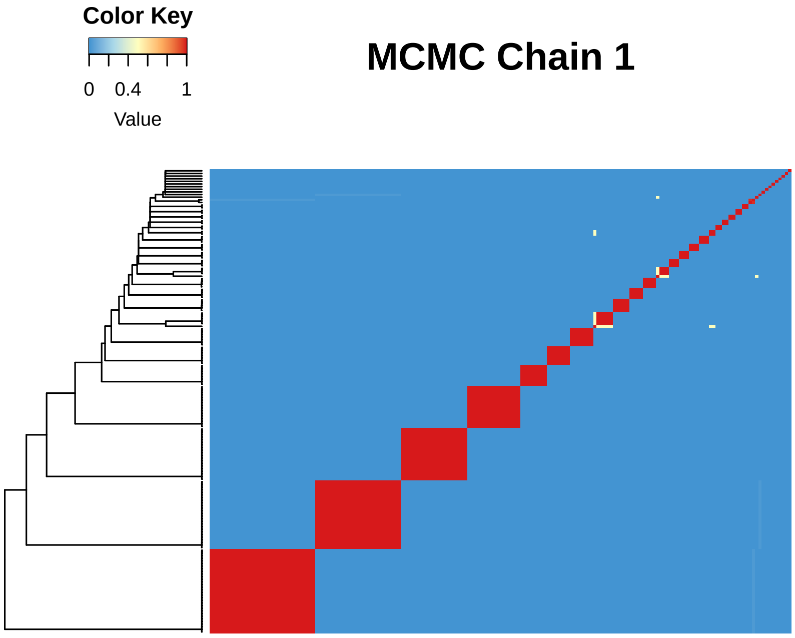

Figure 10: Heatmap visualization of the posterior co-clustering matrix of Gibbs sampling MCMC chain number 1 from the TCGA kidney cancer example. The value in row i and column j is the estimated posterior probability of samples i and j being assigned to the same global cluster.

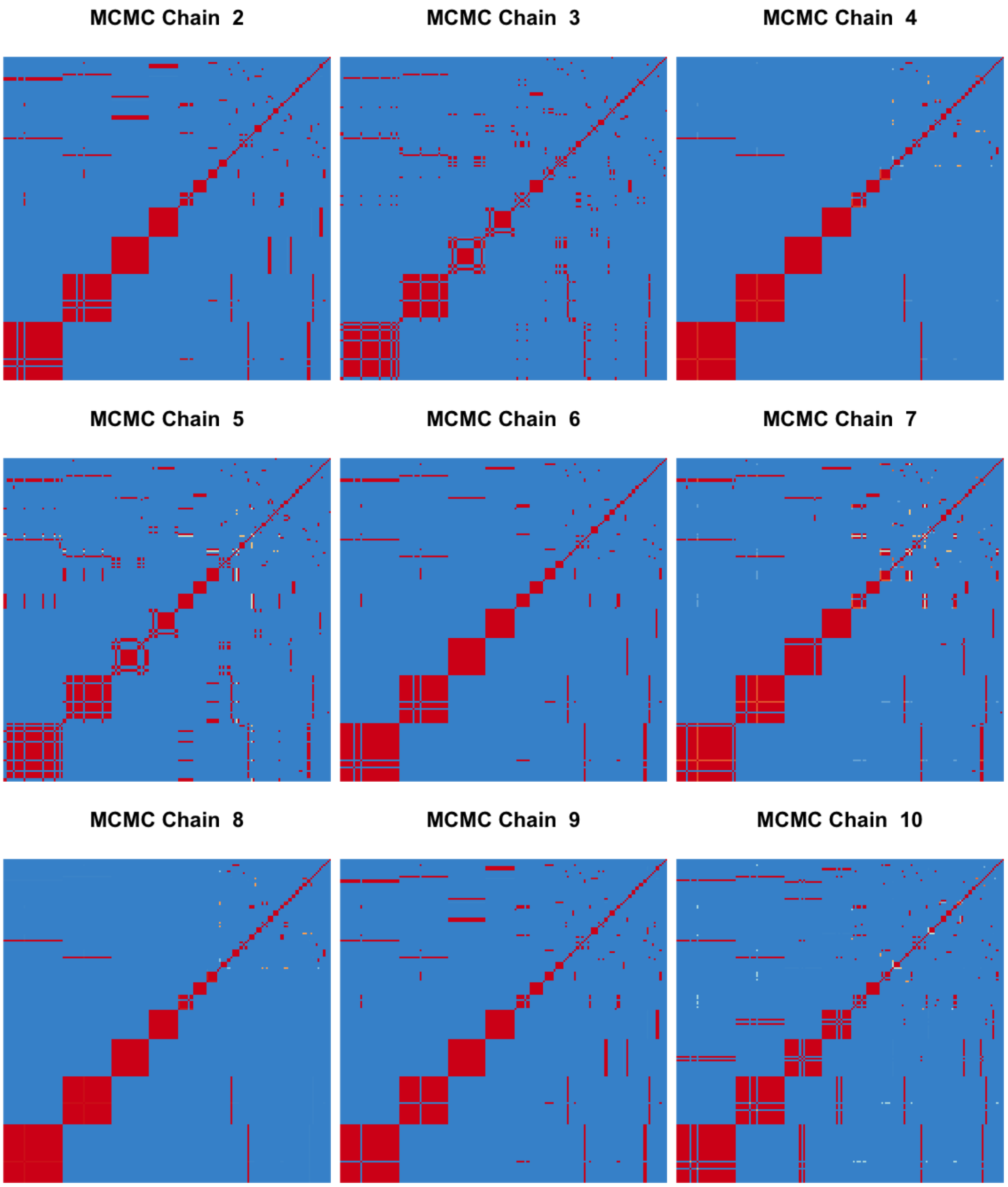

Figure 11: Heatmap visualizations of the posterior co-clustering matrices of Gibbs sampling MCMC chains number 2 to 10 from the TCGA kidney cancer example. The rows and columns have the same order as in Figure 10 to facilitate comparison. The number of context-specific clusters $K^{(c)} = 3$, and the number of global clusters $S = 31$.

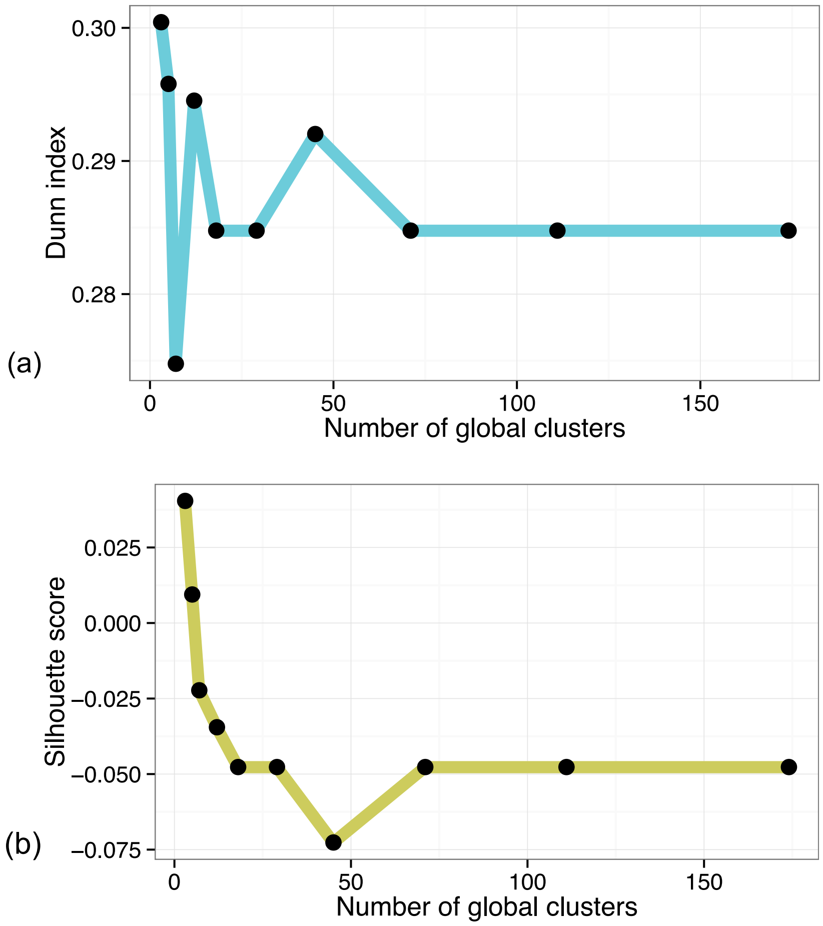

Figure 12: Two commonly used cluster consistency criteria, the Dunn index (a) and the Silhouette score (b), evaluated on clustering results from the Clusternomics model. Both criteria are maximised for compact and consistent clusters. However, in this application the values of both criteria do not agree with the predictive power of the clustering results in terms of survival probabilities.
